# *Myh6*-driven Cre recombinase activates the DNA damage response and the cell cycle in the myocardium in the absence of loxP sites

**DOI:** 10.1242/dmm.046375

**Published:** 2020-12-18

**Authors:** Xinrui Wang, Amelia Lauth, Tina C. Wan, John W. Lough, John A. Auchampach

**Affiliations:** 1 Department of Pharmacology and Toxicology, Medical College of Wisconsin, Milwaukee, WI 53226, USA; 2 Cardiovascular Center, Medical College of Wisconsin, Milwaukee, WI 53226, USA; 3 Department of Cell Biology, Neurobiology and Anatomy, Medical College of Wisconsin, Milwaukee, WI 53226, USA

**Keywords:** Cre recombinase, Transgenic animals, Conditional gene deletion, MerCremer, DNA damage response, Cell cycle, Apoptosis, Fetal marker genes

## Abstract

Regeneration of muscle in the damaged myocardium is a major objective of cardiovascular research, for which purpose many investigators utilize mice containing transgenes encoding Cre recombinase to recombine loxP-flanked target genes. An unfortunate side effect of the Cre-loxP model is the propensity of Cre recombinase to inflict off-target DNA damage, which has been documented in various eukaryotic cell types including cardiomyocytes (CMs). In the heart, reported effects of Cre recombinase include contractile dysfunction, fibrosis, cellular infiltration and induction of the DNA damage response (DDR). During experiments on adult mice containing a widely used *Myh6-merCremer* transgene, the protein product of which is activated by tamoxifen, we observed large, transient, off-target effects of merCremer, some of which have not previously been reported. On Day 3 after the first of three daily tamoxifen injections, immunofluorescent microscopy of heart sections revealed that the presence of merCremer protein in myonuclei was nearly uniform, thereafter diminishing to near extinction by Day 6; during this time, cardiac function was depressed as determined by echocardiography. On Day 5, peaks of apoptosis and expression of DDR-regulatory genes were observed, highlighted by >25-fold increased expression of *Brca1*. Concomitantly, the expression of genes encoding cyclin-A2, cyclin-B2 and cyclin-dependent kinase 1, which regulate the G_2_/S cell-cycle transition, were dramatically increased (>50- to 100-fold). Importantly, immunofluorescent staining revealed that this was accompanied by peaks in Ki67, 5′-bromodeoxyuridine and phosphohistone H3 labeling in non-CMs, as well as CMs. We further document that tamoxifen-induced activation of merCremer exacerbates cardiac dysfunction following myocardial infarction. These findings, when considered in the context of previous reports, indicate that the presence of merCremer in the nucleus induces DNA damage and unscheduled cell-cycle activation. Although these effects are transient, the inclusion of appropriate controls, coupled with an awareness of the defects caused by Cre recombinase, are required to avoid misinterpreting results when using Cre-loxP models for cardiac regeneration studies.

This article has an associated First Person interview with the first author of the paper.

## Abstract (precis)

**Summary:** The presence of tamoxifen-induced Cre recombinase (merCremer) in the nucleus of cardiomyocytes induces DNA damage and unscheduled cell-cycle activation, indicating the requirement for appropriate controls when using Cre-loxP models for cardiac regeneration studies.

## INTRODUCTION

A major objective of the cardiovascular research community is to devise approaches to regenerate the muscular portion of the damaged or diseased myocardium. As recently reviewed, approaches to attain this end include transplantation of induced pluripotent stem cell-derived cardiomyocytes (CMs), trans-differentiation of non-CMs into CMs, and the expansion of pre-existing CMs, which are in a proliferative-senescent state, via cell-cycle activation (Sadek and Olson, 2020). Many of the studies designed to fulfill this objective rely upon experiments utilizing mice, wherein loxP-flanked (i.e. ‘floxed’) target genes are up- or downregulated via genetic recombination, mediated by the bacterial enzyme Cre recombinase. Cre recombinase protein, formats of which confer either constitutive or conditional activation via treatment with tamoxifen, is introduced to floxed animals by mating the latter with mice expressing a Cre recombinase transgene, transcription of which is driven by promoters of genes that are expressed exclusively in CMs. Among these, the promoter driving expression of the α-myosin heavy chain gene – *Myh6* – is a strong ‘Cre driver’ that is widely utilized.

During the past two decades, experiments utilizing constitutive as well as tamoxifen-activated *Myh6*-driven Cre recombinase transgenes have driven remarkable discovery and insight into the roles of many genes involved in all aspects of CM biology. For example, experiments utilizing a tamoxifen-activated transgene termed *Myh6-merCremer* (Sohal et al., 2001), the product of which (merCremer) responds to tamoxifen by translocating from the cytoplasm to the nucleus, have been cited in more than 250 instances according to the PubMed database. However, in addition to targeting and recombining target genes flanked by cognate loxP restriction sites, Cre recombinase has been reported to inflict off-target DNA damage in a loxP-independent fashion in a variety of eukaryotic cell types (Jeannotte et al., 2011; Janbandhu et al., 2014; Schmidt-Supprian and Rajewsky, 2007; Schmidt et al., 2000; Loonstra et al., 2001), including CMs, in which expression of the tamoxifen-activated *Myh6-merCremer* transgene has been shown to cause contractile dysfunction (Koitabashi et al., 2009; Hougen et al., 2010; Bersell et al., 2013; Hall et al., 2011; Lexow et al., 2013), fibrosis (Lexow et al., 2013; Bersell et al., 2013), cellular infiltration (Lexow et al., 2013; Koitabashi et al., 2009; Hall et al., 2011) and DNA damage resulting in cell death (Bersell et al., 2013). Although most defects caused by this transgene are transient, it was recently reported that mice expressing a constitutively active (i.e. tamoxifen-independent) *Myh6-Cre* transgene exhibit cardiac defects that are apparently permanent (Pugach et al., 2015; Gillet et al., 2019). It remains of concern that, despite the potential for off-target effects of Cre recombinase to obfuscate experimental results when using Cre/loxP genetic models, many published reports continue to neglect to include necessary controls.

During a recent study utilizing tamoxifen-induced merCremer to recombine a loxP-flanked target, we observed that loxP-free control mice expressing merCremer exhibited a cell cycle phenotype during the 10-day period immediately following the first of three daily tamoxifen injections. Although these effects were transient, as in the studies cited above, we observed phenomena that included the near-uniform presence of merCremer protein within myonuclei, which receded prior to observations of peak expression in parameters indicating cardiac defects. Of importance, with regard to cardiac regeneration studies, we also observed evidence of remarkably strong cell-cycle activation in non-CMs, as well as in CMs; further, activation of the merCremer transgene impaired functional recovery following myocardial infarction (MI). These and other findings should mandate the inclusion of appropriate controls, and the exercise of caution, when performing and interpreting the results of cardiac regeneration experiments utilizing Cre recombinase to overexpress or delete loxP-flanked genes that regulate the CM cell cycle and subsequent CM proliferation.

## RESULTS

### Experimental scheme and timeline

**Fig. 1.**
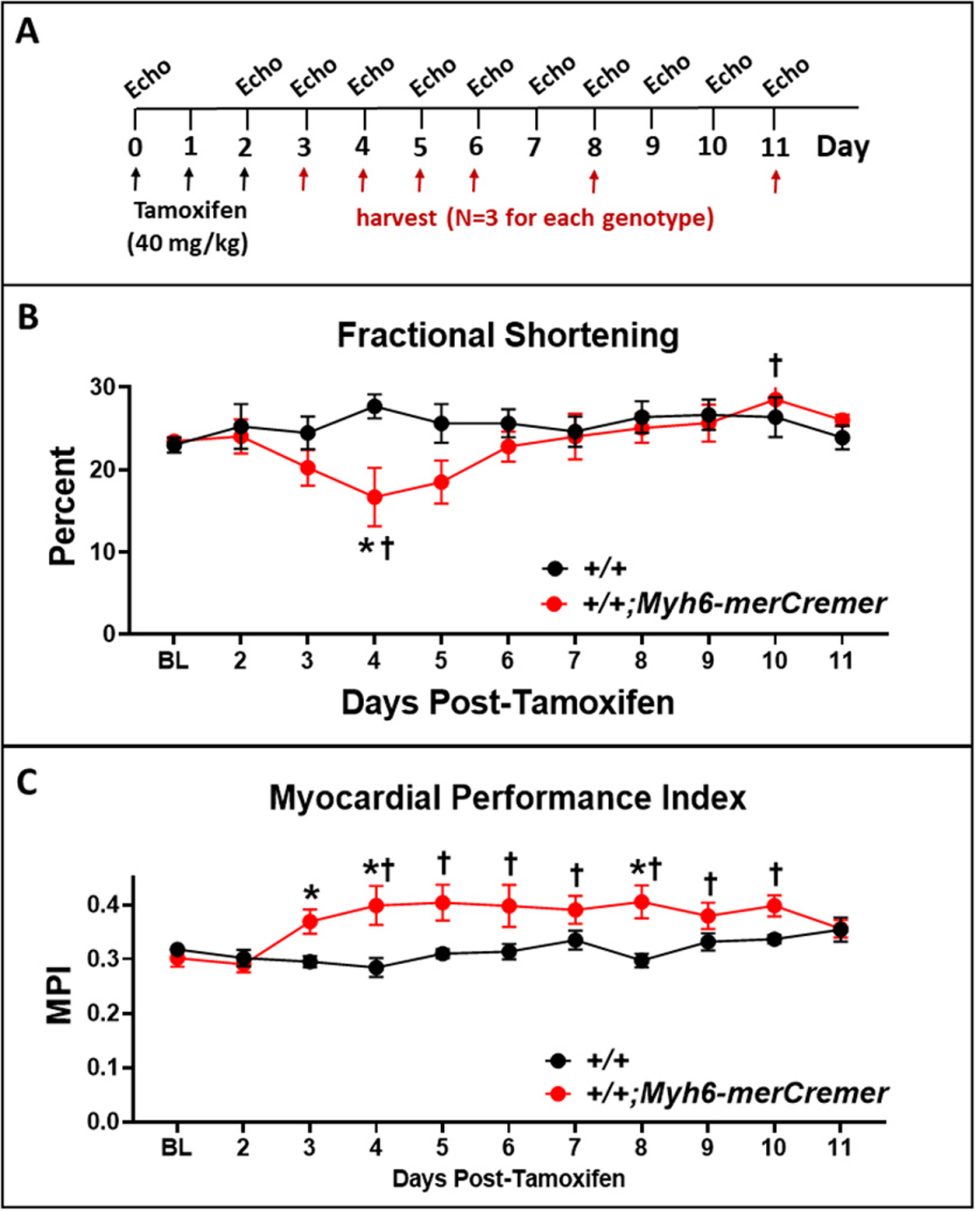
**Echocardiographic data showing that activation of merCremer in naïve mice induces transient cardiac dysfunction.** (A) The experimental timeline. Adult 10- to 14-week-old wild-type (*+/+*) mice (*n*=4), and mice (*n*=6) expressing a tamoxifen-induced Cre recombinase transgene (*+/+;Myh6-merCremer*), were injected with tamoxifen (40 mg/kg) on three consecutive days, followed by echocardiographic assessments of cardiac function, including (B) fractional shortening and (C) the myocardial performance index (MPI). Data are means±s.e.m. (in all figures). *P<0.05 versus +/+; ^†^*P*<0.05 versus baseline value (Day 0). Additional echocardiographic parameters are reported in Fig. S1.

**Fig. 2.**
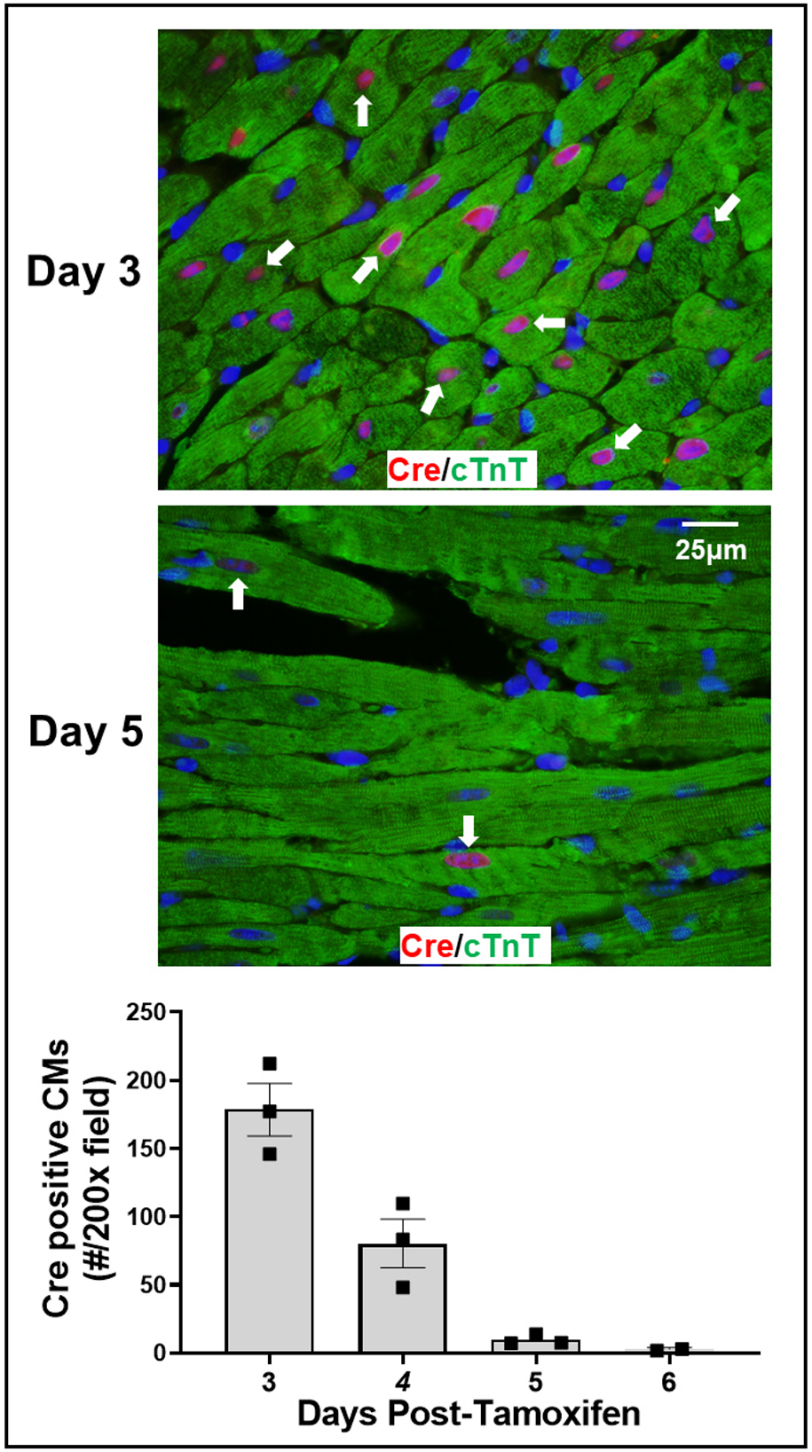
**Immunohistochemical localization of merCremer in cardiomyocytes** **(CMs).** Hearts from mice treated with tamoxifen, as described in Fig. 1, were histologically processed and double-immunostained using antibodies to detect the presence of merCremer in the nucleus, and, to verify CM identity, cardiac troponin T (cTnT) in the cytoplasm. In each heart, a minimum of 1000 CMs were evaluated for nuclear merCremer by examining 200× microscopic images from six randomly selected areas. Arrows point to examples of merCremer-positive CMs.

**Fig. 3.**
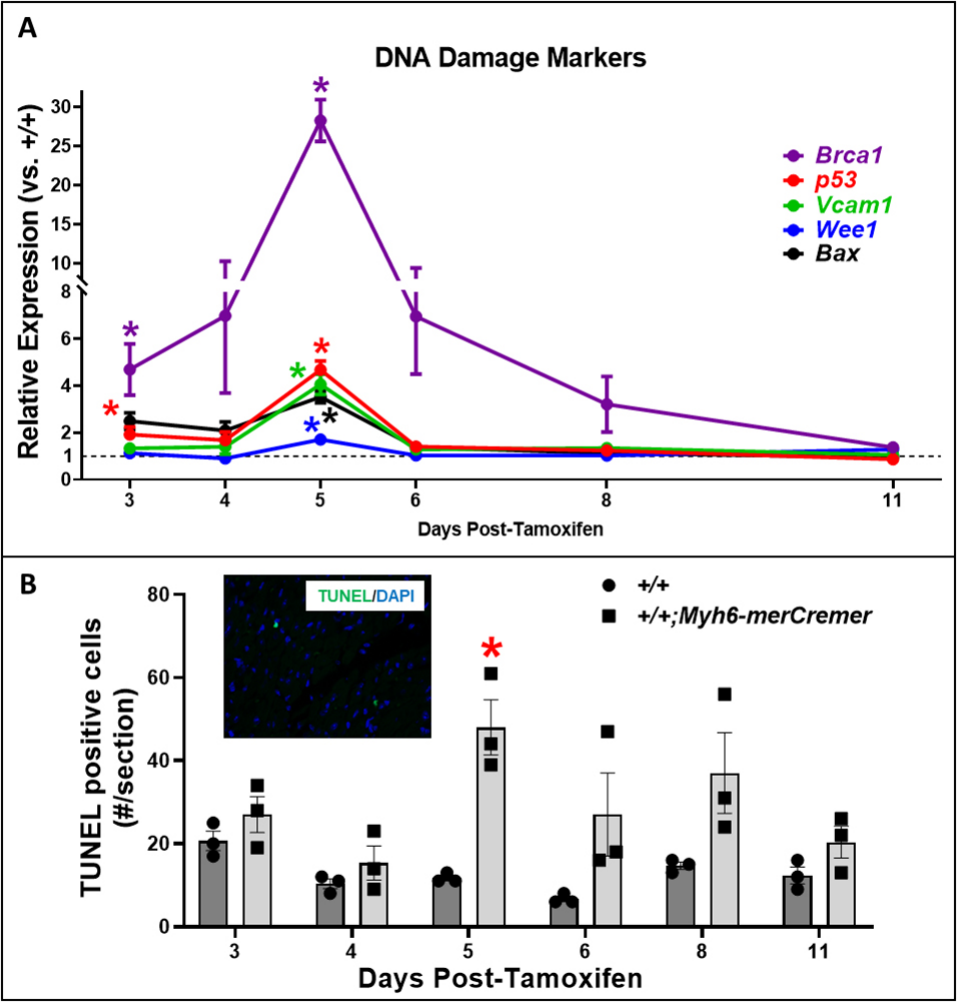
**qRT-PCR determinations indicating DNA damage in hearts after tamoxifen-induced activation of merCremer.** (A) qRT-PCR assessments showing that Cre activation caused increased expression of a battery of genes encoding markers of DNA damage. Data are presented as fold changes relative to +/+ mice, normalized to *Gapdh*. The colored lines denote the expression of each gene in *+/+;Myh6-merCremer* hearts, relative to expression in +/+ wild-type controls at *y*=1 (dashed line), normalized to *Gapdh*. (B) Correlated TUNEL determinations. The inset shows a representative 600× TUNEL image; all TUNEL signals were nuclear (verified by DAPI staining). *n*=3 hearts per time point; *P<0.05 versus +/+.

**Fig. 4.**
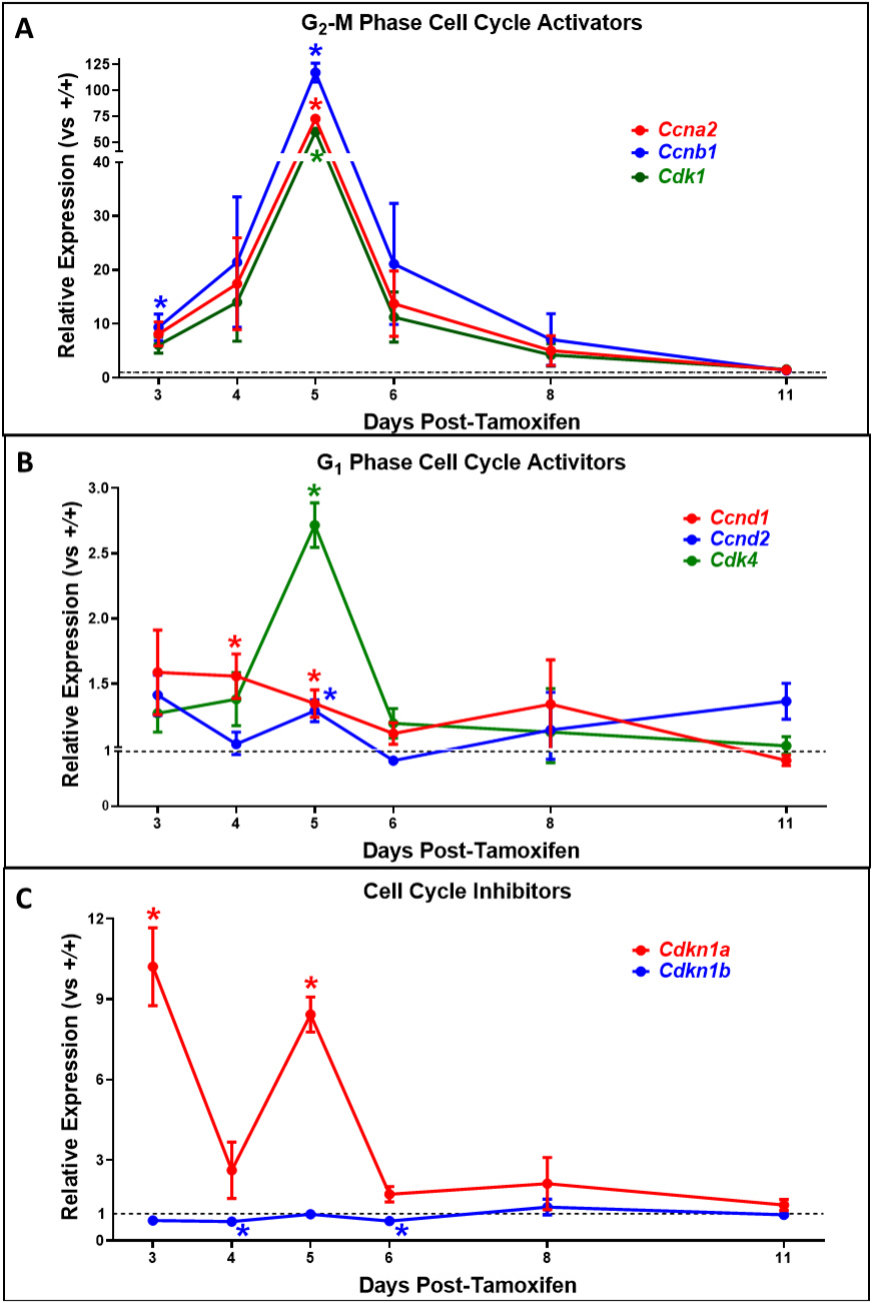
**qRT-PCR determinations showing activation of genes that regulate cell-cycle stage G_2_/M, and the gene encoding p21, in hearts following tamoxifen-induced activation of merCremer.** (A-C) The effect of tamoxifen-activated merCremer on the expression of genes in the heart that activate cell-cycle stages G_2_/M (A) and G_1_ (B), and genes that inhibit the cell cycle (C). The expression of each gene was normalized to *Gapdh*. In each panel, colored lines denote expression of each gene in *+/+;Myh6-merCremer* hearts, relative to expression in +/+ wild-type controls at *y*=1 (dashed lines). *n*=3 hearts per time point; *P<0.05 versus +/+.

**Fig. 5.**
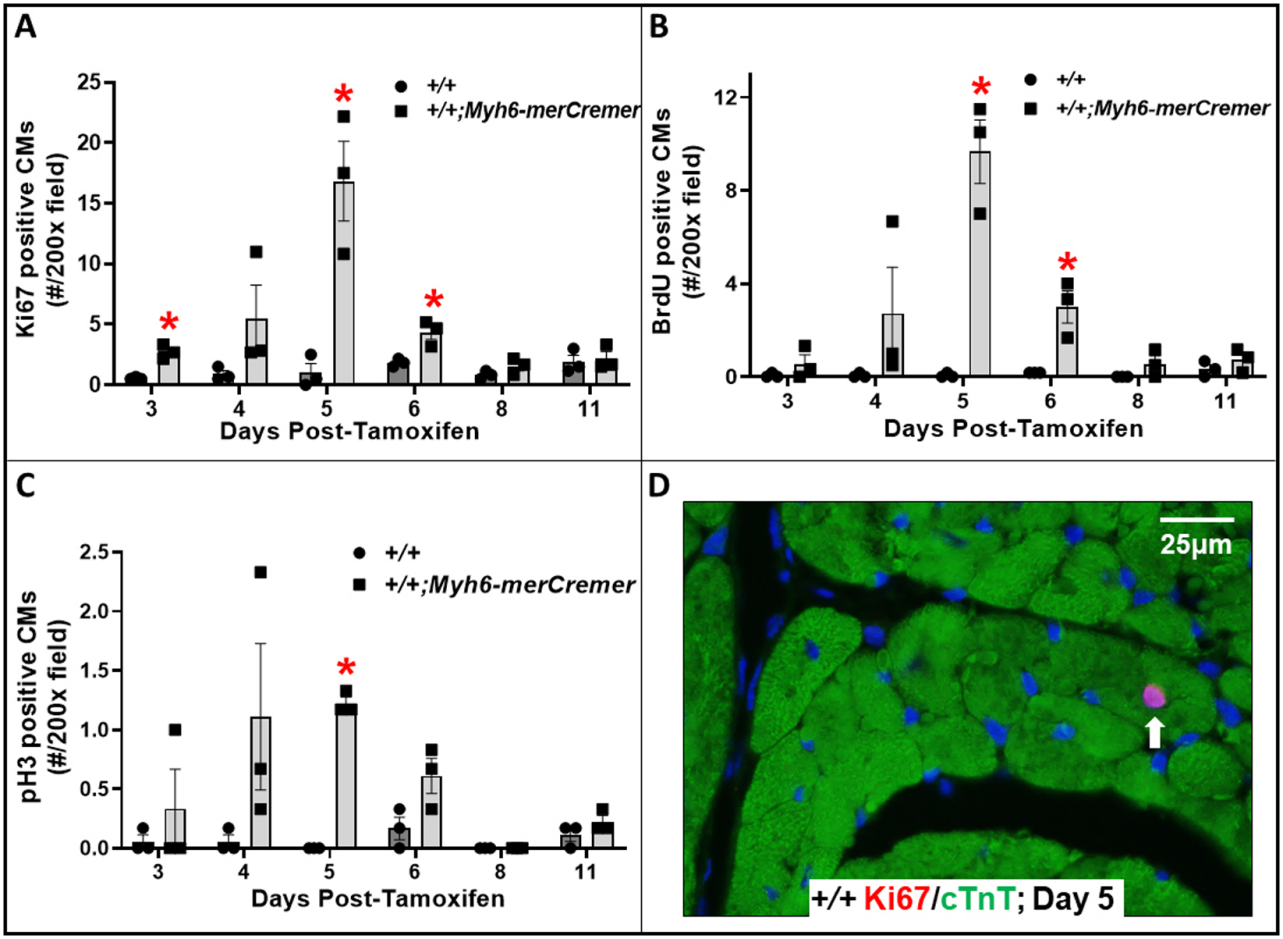
**merCremer activates the cell cycle in** **CMs.** (A-C) The effect of tamoxifen-activated merCremer on numbers of CMs exhibiting cell-cycle activation, as indicated by the nuclear presence of Ki67 (A), BrdU (B) and pH3 (C). (D) A typical example of a Ki67-positive CM nucleus (arrow). *n*=3 hearts per time point; *P<0.05 versus +/+.

**Fig. 6.**
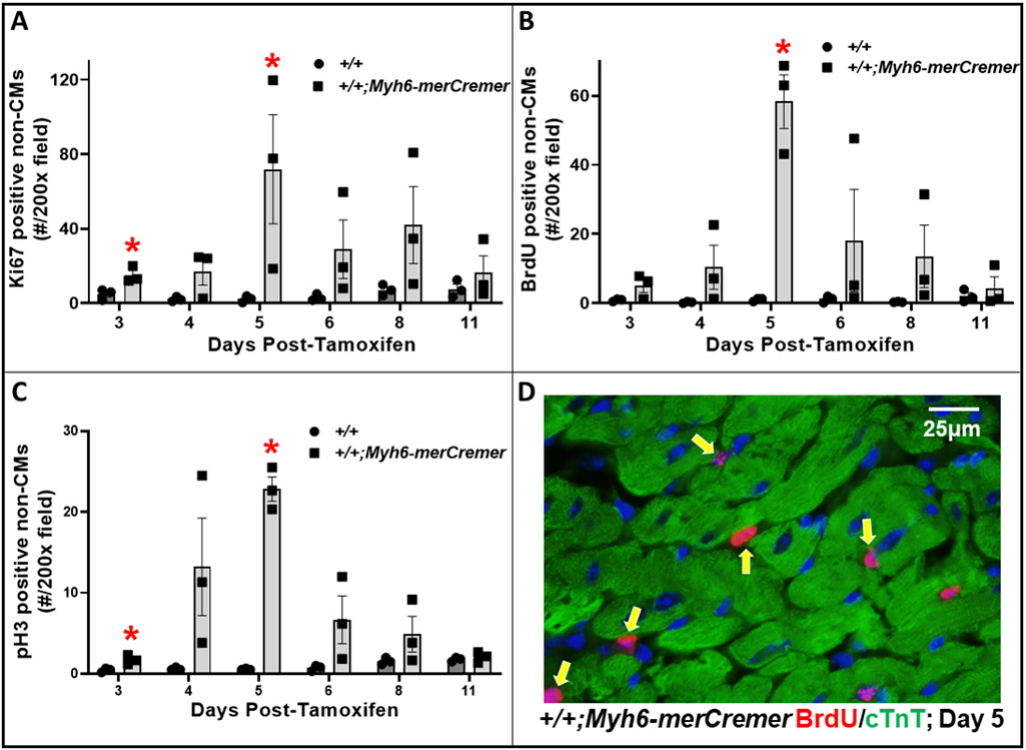
**merCremer activates the cell cycle in non-cardiomyocytes.** (A-C) The effect of tamoxifen-activated merCremer on numbers of non-CMs exhibiting cell-cycle activation, as indicated by the nuclear presence of Ki67 (A), BrdU (B) and pH3 (C). (D) Typical examples of BrdU-positive nuclei in non-CMs (arrows). *n*=3 hearts per time point; *P<0.05 versus +/+.

These experiments were designed to examine the off-target effects of tamoxifen-activated Cre recombinase (merCremer) in CMs of adult mouse hearts that do not possess loxP restriction sites. We evaluated the effects of treating 10- to 14-week-old littermates, possessive of wild-type (designated *+/+*) and *Myh6-merCremer* (designated *+/+;Myh6-merCremer*) genotypes, with a single dose of tamoxifen (40 mg/kg) on three consecutive days (Bersell et al., 2013; Hall et al., 2011; Hougen et al., 2010; Khalil et al., 2019; Koitabashi et al., 2009; Leach et al., 2017; Lexow et al., 2013; Tane et al., 2014; Xiang et al., 2016). Because both genotypes were identically treated with tamoxifen, the only variable tested was the presence or absence of merCremer; the effects of tamoxifen alone, which in the absence of merCremer have been shown to have no effect (Hall et al., 2011; Bersell et al., 2013; Lexow et al., 2013), were not examined here. Fig. 1A presents the experimental timeline, during which hearts were monitored for cardiac function, localization of merCremer protein in the nucleus, DNA damage response (DDR) and cell-cycle activation for 11 days following the first tamoxifen injection on Day 0; this timeline is denoted ‘Days Post-Tamoxifen’ in Figs 1–6.

### Tamoxifen-induced activation of merCremer causes transient cardiac dysfunction

Fig. 1B and C show that tamoxifen-induced activation of merCremer caused cardiac dysfunction, as determined by echocardiography, revealing decreases in fractional shortening (Fig. 1B) and increases in the myocardial performance index (MPI; Fig. 1C); an increase in the MPI or Tei index, wherein the summation of the isovolumic contraction and relaxation times is increased relative to ejection, estimates the presence of global systolic and/or diastolic dysfunction (Broberg et al., 2003; Tei et al., 1995; Poulsen et al., 2000; Schaefer et al., 2005). Dysfunction began to appear on Day 3 after the first tamoxifen injection. Although the effect on fractional shortening was most pronounced on Day 4, when a ∼40% decline was observed, this parameter became normalized by Day 6; by contrast, the effect on MPI, with increases ranging from 15% to 30% during Days 3-10, persisted until Day 11. Hence, in accord with the previous findings cited in the Introduction, tamoxifen-induced merCremer caused transient cardiac dysfunction. Additional echocardiographic parameters, which further revealed transient thickening of the left ventricular walls beginning on Day 6, are reported in Fig. S1.

### Tamoxifen induces the transient presence of merCremer in cardiomyocyte nuclei

Because merCremer protein translocates into the nucleus upon binding to the modified estrogen receptor (mer) motifs of the merCremer protein to cause activation, it was of interest to monitor the temporal presence of nuclear merCremer in response to tamoxifen administration, relative to its off-target effects. As shown in Fig. 2, immunofluorescent staining of merCremer protein on successive days following tamoxifen administration revealed its presence in nearly all CM nuclei on the third day (Day 3) after the first tamoxifen injection, which declined thereafter to insignificant levels by Day 6, in correlation with reduced fractional shortening (Fig. 1B).

### Activation of merCremer induces mRNA expression of DDR, inflammation and apoptosis markers

Based on a report that levels of the DNA damage marker γH2A.X are increased in hearts expressing this *Myh6-merCremer* transgene (Bersell et al., 2013), we performed real-time PCR to assess the extent to which merCremer affects the level of mRNAs encoding the DDR, inflammation and apoptosis, as shown in Fig. 3A. Data are presented as fold changes relative to +/+ normalized to *Gapdh* at each time point. This revealed increased expression of all selected markers as early as Day 3, followed by simultaneous peaks of expression on Day 5. Although these assessments interrogated gene expression changes in all cells contained in the heart tissue samples employed for quantitative reverse transcription PCR (qRT-PCR), it was interesting to note that the peak of expression on Day 5 occurred 2 days after the near-uniform occupancy of merCremer protein in CM nuclei seen on Day 3 (Fig. 3). Most remarkably, the DDR marker *Brca1* exhibited a ∼30-fold increase on Day 5, while other markers peaked <5-fold. Notably, the apoptosis marker *Bax* increased 3.5-fold on Day 5, in correlation with the peak in apoptosis indicated by the presence of terminal deoxynucleotidyl transferase dUTP nick end labeling (TUNEL) fluorescent signal in myocardial nuclei, which might represent CMs and/or non-CMs (Fig. 3B). Table S1 presents ΔCq values of the target genes normalized to *Gapdh*.

### Activation of merCremer strongly induces G_2_/M cell-cycle-regulatory genes

Currently, mouse models containing transgenes that express Cre recombinase in a CM-specific fashion are in widespread use to manipulate the expression of loxP-flanked genes that regulate the CM cell cycle. Because potential effects of Cre recombinase alone on CM cell-cycle activity have not previously been reported, the assessments shown in Figs 4 and 5 were performed. Fig. 4 shows results of real-time PCR determinations to assess the effect of merCremer activation on expression of cell-cycle-regulatory genes during the Day 3-11 experimental timeline. Although these determinations may reflect changes occurring in myocardial interstitial cells in addition to CMs, it was remarkable that the expression of genes that regulate cell-cycle phase G_2_/M – *Ccna2* (cyclin-A2), *Ccnb1* (cyclin-B1) and *Cdk1* (cyclin-dependent kinase 1) – was significantly upregulated as early as Day 3 (Fig. 4A), while increases in expression of the G_1_ phase regulators *Ccnd1* (cyclin-D1) and *Cdk4* (cyclin-dependent kinase 4) were relatively modest (Fig. 4B). Expression of the gene encoding cell-cycle inhibitor p21 (*Cdkn1a*) was significantly increased (10-fold) at Day 3 (Fig. 4C). These phenomena were followed on Day 5 by an enormous increase in expression of the G_2_/M cell-cycle-regulator genes, ranging from 60-fold (*Cdk1*) to 117-fold (*Ccnab1*); although a trend toward increased expression of the G_1_ regulators was also observed on Day 5, with the exception of *Cdk4* these increases were non-significant. Also, as on Day 3, expression of the gene encoding p21 was strongly increased on Day 5 (>8-fold). These observations were followed by precipitous declines in expression of all the genes 1 day later (post-tamoxifen Day 6), followed by declining expression at later time points.

In addition to the genes evaluated in Fig. 4, we also monitored the expression of *Osm*, *Osmr*, *Runx1* and *Myh7* in response to merCremer activation, because these genes are regarded as de-differentiation markers that become expressed prefatory to CM cell-cycle activation (Kubin et al., 2011). In addition, we assessed *Acta1*, *Acta2*, *Myh6*, *Myh7*, *Nppa* and *Nppb*, because these fetal genes are re-expressed during CM hypertrophy. As shown in Fig. S2, expression of the de-differentiation genes was increased as early as Day 3, followed by peaks of significantly increased expression on Days 5 and 6. Similar to the genes monitored in Fig. 4, expression of the de-differentiation markers subsided on Day 6, although increased expression of *Myh7*, which is also regarded as a marker of fetal gene expression (Cox and Marsh, 2014), was sustained. As shown in Fig. S3, expression of other fetal genes was also elevated 3-8 days post-tamoxifen treatment, with the exception of *Myh6*, which was reciprocally reduced relative to *Myh7*.

### Activation of merCremer induces cell-cycle activation in cardiomyocytes and in non-cardiomyocytes

The pattern of increased levels of pro-proliferative cell-cycle mRNAs observed in merCremer-activated hearts on Days 4-6, which peaked at Day 5, coincided with the pattern of increased numbers of CMs expressing cell-cycle activation markers Ki67 (also known as Mki67) (Fig. 5A,D), 5′-bromodeoxyuridine (BrdU; Fig. 5B) and phosphohistone H3 (pH3; Fig. 5C). Considering that *Myh6-merCremer* is expressed only in CMs, it was somewhat surprising that a similar pattern of cell-cycle activation was observed in non-CMs (Fig. 6), numbers of which were ∼3-5-fold in excess of those observed in CMs.

### Dysfunction caused by myocardial infarction is exacerbated by merCremer activation

**Fig. 7.**
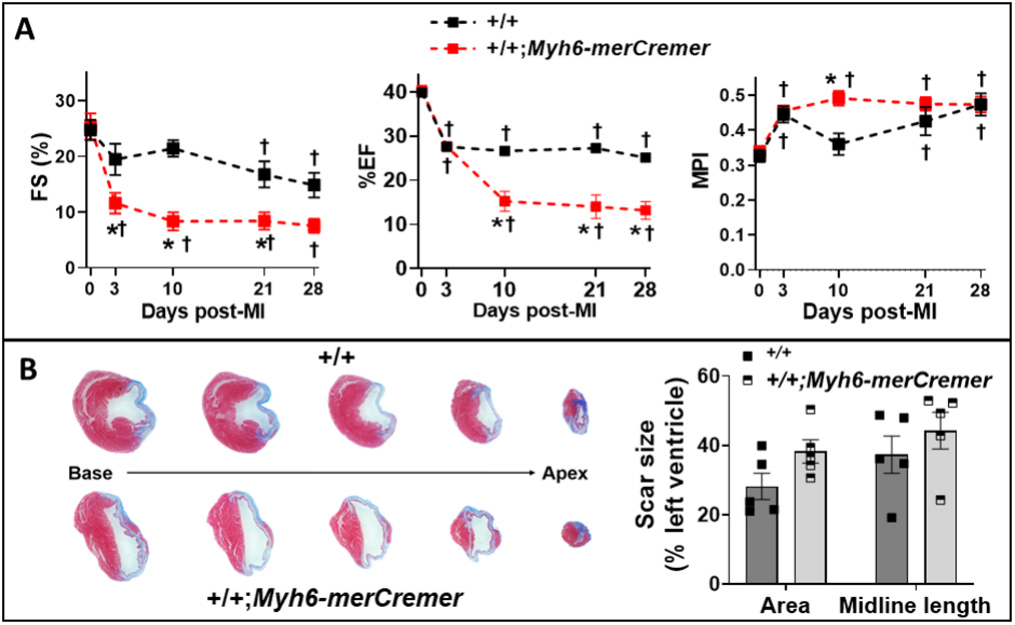
**merCremer increases cardiac dysfunction following myocardial infarction (MI).** Wild-type (*+/+*) and *+/+;Myh6-merCremer* mice were subjected to MI, followed 3 days later by tamoxifen-induced activation of merCremer (40 mg/kg/day×3 days). (A) Indices of left ventricular function [fractional shortening (FS), ejection fraction (EF) and MPI; *n*=5/group] determined by echocardiography on the indicated days post-MI. Additional echocardiographic parameters are provided in Table S2. (B) Representative trichrome-stained cross-sections (left) obtained at intervals of 0.8 mm along the basal-apical axis of +/+ and +/+*;Myh6-merCremer* hearts at 28 days post-MI; blue stain denotes the area of the scar. Scar size was quantified (right) by measurement of the area and midline length. *P<0.05 versus +/+; ^†^*P*<0.05 versus baseline value (0 days post-MI).

The *Myh6-merCremer* transgene model employed here is widely used in cardiac regeneration studies to assess the effects of altering the expression of loxP-targeted genes after cardiac injury. It was therefore of interest to ascertain whether tamoxifen-activated merCremer, in the absence of loxP sites, affected general parameters that are commonly monitored in regeneration studies following experimental MI. Hearts in wild-type (*+/+*) and *+/+;Myh6-merCremer* mice were infarcted as described in the Materials and Methods, followed by injection with 40 mg/kg tamoxifen for three consecutive days, beginning on the third day post-MI. During this timeline, echocardiography was performed at selected intervals up to the 28-day post-MI time point, when hearts were removed for scar size analysis. Echocardiography revealed that activation of merCremer exacerbated the extent of cardiac dysfunction caused by MI at all time points (Fig. 7A; Table S4), which was accompanied by a non-significant trend toward increased scar formation (Fig. 7B).

## DISCUSSION

These findings show that, consequent to conditionally activating the *Myh6*-driven *merCremer* transgene in wild-type CMs via intraperitoneal administration of 40 mg/kg tamoxifen for three consecutive days, cardiac dysfunction occurs (Fig. 1), concomitant with induction of the DDR (Fig. 3A), increased apoptosis (Fig. 3B) and activation of the cell cycle (Fig. 4) in CMs (Fig. 5) as well as in non-CMs (Fig. 6). These phenomena, except for apoptosis, peaked on Day 5 after the first tamoxifen injection, which followed the near-uniform occupancy of merCremer protein in myonuclei on Day 3 (Fig. 2). The effects of merCremer were transient in naïve mice, approaching wild-type levels by Day 8 and complete resolution by Day 11. When activated in the setting of MI, however, merCremer produced an exacerbation of cardiac dysfunction that was sustained for at least 28 days (Fig. 7). Regarding the effect on CMs, although it is unlikely that new CMs could be generated resultant from this brief transient response, the likelihood of cardiac hypertrophy, which would be one interpretation of the increased expression of fetal genes observed beginning on Day 5 (Figs S2 and S3), should be considered. The experimental design employed here focused on the effects of merCremer alone because several studies have shown, despite a report to the contrary (Koitabashi et al., 2009), that tamoxifen alone does not affect the myocardium (Lexow et al., 2013; Bersell et al., 2013; Hall et al., 2011). And, although we have not formally assessed whether the presence of loxP sites in the genome could mitigate the off-target effects of merCremer as previously suggested (Silver and Livingston, 2001), this is deemed unlikely based on our observations using adult mice containing loxP-flanked *Kat5* alleles (X.W., T.C.W. and A.L. et al., unpublished observations).

Our observations that merCremer induces transient cardiac dysfunction accompanied by the induction of DDR-regulatory genes and apoptosis in the myocardium confirm previous reports (Koitabashi et al., 2009; Hall et al., 2011; Lexow et al., 2013; Hougen et al., 2010; Bersell et al., 2013). To our knowledge, however, effects of Cre recombinase on cell-cycle activation in CMs (Fig. 5) and in non-CMs (Fig. 6) have not previously been reported. In this regard, the peaks of G_2_/M regulatory gene activation seen on Day 5 (Fig. 4A), which could be attributed to alterations in CMs and/or non-CMs, might be unprecedented in terms of fold induction levels. This observation is similar to findings wherein depletion of glycogen synthase kinase-3 (Zhou et al., 2016) or overexpression of cyclin D1 induced by merCremer in CMs (Tane et al., 2014) dramatically increased the expression of G_2_/M cell-cycle-regulatory genes, as well as p21 mRNA and protein levels. These effects culminated in G_2_ blockade, an effect of Cre recombinase described in other cell types (Janbandhu et al., 2014).

Regarding the effect of merCremer on non-CMs, this was surprising because expression driven by the *Myh6* promoter is CM specific. We speculate that this reflects a paracrine-induced inflammatory response, a possibility indicated by increased expression of *Vcam1* (Fig. 3), as well as by reports of cellular infiltration into the myocardium in response to activation of merCremer (Koitabashi et al., 2009; Hall et al., 2011; Lexow et al., 2013). It is also possible that cell-cycle activation in non-CMs is caused by compensated changes resultant from the cardiac dysfunction described in Fig. 1. Because cardiac dysfunction appears 1-2 days before the peaks in DDR marker expression, apoptosis and cell-cycle activation seen on Day 5, it is possible that these defects result from, but are not the source of, cardiac dysfunction. It is also possible that the extent of DNA damage caused by the presence of merCremer in myonuclei at or prior to Day 3, when significantly increased expression of genes encoding Brca1, p53, cyclin-B1 and p21, as well as increased cell-cycle activation in CMs (Ki67) and non-CMs (Ki67, pH3) is detected, is sufficient to cause cardiac dysfunction, which in turn may exacerbate the expression of these markers at later stages.

The chronology seen in Figs 3-5, showing the near-uniform occupancy of merCremer protein in myonuclei on Day 3, which receded thereafter and was followed by peaks of DNA damage, apoptosis and cell-cycle activation, supports the likelihood that these effects were initiated by the transient localization of merCremer in myonuclei (Lexow et al., 2013). Although these defects, which followed tamoxifen-induced activation of merCremer, were transient, this observation argues for caution when using constitutively active Cre recombinase models, because, as we previously observed (Fisher et al., 2016), constitutively active Cre recombinase appears to be retained within myocyte nuclei. In this regard, it was recently reported that *Myh6*-driven expression of constitutive (tamoxifen-independent) Cre recombinase in the hearts of wild-type mice disrupts the *Dmd* gene encoding dystrophin (Gillet et al., 2019), which is interesting because the *Dmd* gene contains a degenerate loxP site (Pugach et al., 2015). The study by Pugach et al. (2015) is noteworthy because it describes degenerate loxP sites in 227 genes (including *Dmd*) of the C57Bl/6 genome, among which 55 are expressed in cardiac muscle. Taken together, these findings indicate that studies employing constitutively active Cre recombinase, or tamoxifen-induced merCremer, to recombine loxP-flanked target genes, which are in widespread use in studies designed to promote CM proliferation following cardiac damage, should anticipate the occurrence of off-target effects, and be appropriately controlled.

## MATERIALS AND METHODS

### Animal care and use

This investigation adhered to the National Institutes of Health (NIH) Guide for the Care and Use of Laboratory Animals (NIH Pub. Nos. 85-23, Revised 1996). All protocols are described in the authors' Animal Use Application (AUA #225), which was approved by the Medical College of Wisconsin Institutional Animal Care and Use Committee (IACUC), which has Animal Welfare Assurance status from the Office of Laboratory Welfare (A3102-01). In these experiments, C57BL/6 wild-type mice were mated with a transgenic line obtained from The Jackson Laboratory (JAX #005650), which expresses an *α-MHC-merCremer* (*Myh6-merCremer*) transgene encoding merCremer recombinase (Sohal et al., 2001). This transgene is expressed exclusively in CMs, wherein its protein product resides in the cytoplasm. Upon treatment with tamoxifen, merCremer is activated via its translocation into the nucleus, wherein it recombines genes that have been engineered to contain cognate loxP restriction sites. In this study, the effects of merCremer were determined by comparing wild-type (+/+) mice with mice of the same background containing the *Myh6-merCremer* transgene; because both genotypes were identically treated with tamoxifen, the effects, if any, of tamoxifen alone were not examined.

Genotyping was performed by PCR in 20 µl reactions consisting of GoTaq Green Mastermix (Promega #M7123), 1.1 mM MgCl_2_, 0.5 µM each primer, 0.5 μM internal control primers and 4.0 µl template. Templates consisted of 1200 g supernatants of ear samples that had been boiled for 10 min in 0.3 ml 10 mM NaOH/1 mM EDTA. Primer sequences, and the program used to amplify PCR products, are listed in Table S3. Amplicons were separated at 100 V in 2% agarose and imaged by ethidium bromide staining.

For these determinations, equal numbers of adult 10- to 14-week-old wild-type male and female mice on a Bl6/Sv129 background, designated (+/+), and littermates expressing the *Myh6-merCreMer* transgene, designated (+/+*;Myh6-merCremer*), were treated with 40 mg/kg tamoxifen by intraperitoneal injection on three consecutive days (Bersell et al., 2013; Hall et al., 2011; Hougen et al., 2010; Khalil et al., 2019; Koitabashi et al., 2009; Leach et al., 2017; Lexow et al., 2013; Tane et al., 2014; Xiang et al., 2016). Tamoxifen (Sigma-Aldrich #T5648) was suspended in 5% ethanol/sunflower oil and injected intraperitoneally.

### MI

To induce MI, mice were respirated (model 845, Harvard Apparatus) via an endotracheal tube with room air supplemented with 100% oxygen to maintain blood gases within normal physiological limits. The electrocardiogram (ECG; limb lead II configuration) was continuously recorded (Powerlab) using needle electrodes, and rectal temperature was maintained at 37°C throughout the experiments using a servo-controlled heating pad. When anesthetized, thoracotomy was performed to the left of the sternum to expose the heart, followed by opening of the pericardium and placement of an 8.0 nylon suture beneath the left main coronary artery at a level below the tip of the left atrium to target the lower half of the ventricle, with the aid of a microscope. Ischemia was induced by carefully tying the suture with a double knot, after which coronary occlusion was verified by visual observation of blanching of the myocardium distal to the ligature and by ST segment elevation on the ECG. After ligation, the chest wall was closed with polypropylene suture and recovery was monitored until mice became fully ambulatory. Immediately prior to initiating the surgical procedure to produce MI, mice were injected subcutaneously with sustained release meloxicam (4 mg/kg) to limit post-operative pain.

### Echocardiography

Mice were lightly anesthetized with isoflurane delivered via a nose cone. Parasternal long-axis, short-axis and apical four-chamber views were recorded using a VisualSonics Vevo 3100 high-frequency ultrasound system with a transducer (MX550D), operating at 30-40 mHz. Parasternal short-axis views in M-mode were used to measure left ventricular (LV) internal diameter (LVID), anterior wall thickness (LVAW) and posterior wall thickness (LVPW) at end-diastole (d) and end-systole (s). LV systolic function was assessed by fractional shortening {FS (%)=[(LVIDd – LVIDs)/LVIDd]×100}. Long-axis views in B-mode were used to measure left ventricular internal area (LVA) and length (L) at end-diastole and end-systole. LV systolic function was assessed by (1) FS and (2) ejection fraction [EF (%)=(end-diastolic volume – end-systolic volume)/end-diastolic volume], whereby volumes were estimated by 4π/3×L/2×[LVA/π(L/2)]^2^. In addition, global LV function was monitored using the myocardial performance index (MPI=isovolumic contraction time+isovolumic relaxation time/ejection time), which has been shown to be a reliable and reproducible parameter for evaluating LV contractile dysfunction; studies have documented that MPI is independent of heart rate, arterial pressure and preload (Broberg et al., 2003; Tei et al., 1995; Poulsen et al., 2000; Schaefer et al., 2005). Time intervals were obtained from pulsed Doppler waveforms of mitral valve inflow and aortic valve outflow.

On the day before harvest, mice were injected with BrdU (1 mg). At harvest, mice were euthanized with CO_2_ and hearts were immediately perfused with ∼5 ml 25 mM KCl/5% dextrose/PBS (cardioplegic solution). After removal of atria, ventricular samples were apportioned for gene expression analysis by storage in TRIzol (Thermo Fisher Scientific #15591626) at −80^o^ C and for histology by overnight fixation in 4% paraformaldehyde, followed by brief storage in 70% EtOH prior to embedding in paraffin.

### qRT-PCR

Heart tissue in Trizol was purified using PureLink RNA Mini-Kits (Invitrogen #12183018A), including a genomic DNA removal step (PureLink DNase; Thermo Fisher Scientific #12185-010) according to the manufacturer's instructions. RNA yield and quality were determined using an Eppendorf Biophotometer Plus Instrument. Complementary DNA (cDNA) was synthesized in 20 µl reactions containing 1.0 µg total RNA template in 14 µl nuclease-free distilled water (NFDW), 4 µl 5× VILO reaction mix (Invitrogen #100002277) and 2 µl 10× SuperScript Enzyme Mix (Invitrogen #100002279). Reactions were incubated for 10 min at 25°C, 60 min at 42°C, and 5 min at 85°C. Synthesized cDNA was diluted in NFDW to a concentration of 3.125 ng/µl and stored at −20°C until use. qRT-PCR was performed on each biological replicate (i.e. each heart) in triplicate. qRT-PCR reactions were performed in 96-well or 384-well arrays using Taqman Fast-Advanced Master Mix (Thermo Fisher Scientific #4444557), Taqman Probe Kits (Table 1) and 12.5 ng cDNA as template. Arrayed samples were amplified in a Bio-Rad Real-Time PCR System programmed as follows: 2 min at 50°C→0:20 min at 95°C→0:03 min at 95°C→0:30 min at 60°C; the last two steps were repeated 39 times. Results were processed using Bio-Rad CFX Manager 3.1 software.

### Immunostaining

On the day before harvest, mice were injected with BrdU as described above. Following removal, hearts were perfused with cardioplegic solution and atria were removed. Ventricles were fixed overnight in fresh 4% paraformaldehyde/PBS, processed through an EtOH series and embedded in paraffin. Sections (4 µm) mounted on microscope slides were de-waxed, subjected to antigen retrieval (100°C in 10 mM trisodium citrate pH6.0/0.05% Tween 20 for 20 min), followed by 30 min cooling at room temperature, and blocked with 2% goat serum/0.1% Triton X-100 in PBS. Primary antibodies were diluted in blocking buffer and applied overnight at 4°C, and secondary antibodies were applied for 1 h in the dark. Combinations of primary and secondary antibodies employed for each antigen, plus dilutions, are shown in Table S4.

### Quantitative assessment of myocardial scarring

Paraffinized hearts were transversely sectioned, in entirety, from apex to base, after which eight 4 µm-thick sections from equidistant (∼0.8 mm) intervals were placed on microscope slides. The slides were stained with Masson trichrome to quantitatively assess scar size (Leach et al., 2017). Briefly, trichrome-stained sections were examined with a Nikon SMZ800 microscope and photographed at 10× magnification using a SPOT Insight camera (Nikon Instruments). MIQuant software was used to quantitate infarct size in sections between the apex and the ligation suture site, as previously described (Leach et al., 2017). Results were expressed as the average percentage of area and midline length around the left ventricle.

### Quantitative assessment of cell-cycle activation

Immunostains employed to assess cell-cycle activation were Ki67, pH3 and BrdU. To label cells with BrdU, mice were intraperitoneally injected with 1 mg BrdU resuspended in 0.1 ml PBS, 24 h prior to harvest. CM identity was determined by counterstaining cardiac troponin T (cTnT). Fluorescent signals were photographed in six randomly selected fields of the left ventricle at the magnifications listed in figure legends using a Nikon Eclipse 50i microscope equipped with a Nikon DSU3 digital camera. To quantify CM cell-cycle activation, myonuclei (≥1.5 µm diameter) exhibiting signal comprising >50% of the nuclear area and confirmed to be surrounded by cTnT-positive cytoplasm were enumerated in each field by a blinded observer; cells in which nuclei did not conform to these standards were identified as non-CMs. A minimum of 1000 CMs were evaluated in each section (heart). Results are presented as the average number of events per field.

### TUNEL labeling and counting

Apoptosis was assessed using the DeadEnd Fluorometric TUNEL System (Promega #G3250), precisely adhering to the manufacturer's instructions. The total number of TUNEL-positive nuclei in each section was manually counted at 400× magnification. TUNEL signal was counted only if confined to a 4′,6-diamidino-2-phenylindole (DAPI)-positive nucleus. Nuclei were scored as TUNEL-positive only if at least 50% of the nucleus contained fluorescent signal. Despite our attempts to adjust conditions of proteinase-K digestion to enable co-staining of markers of CM identity, these were unfortunately not successful; thus, TUNEL staining described in this paper cannot be ascribed to any specific cell type.

### Statistics

All data are presented as means±s.e.m. Echocardiography data were analyzed by a two-way repeated measures ANOVA (time and genotype) to determine whether there was a main effect of time, genotype or a time-genotype interaction. If global tests showed an effect, post hoc contrasts between baseline and subsequent time points within experimental groups were compared by a Dunnett's multiple comparison *t*-test; differences between genotypes at each time point were compared by a Student's *t*-test with the Bonferroni correction. All other data were compared by an unpaired, two-tailed Student's *t*-test. *P*<0.05 was considered significant.

## Supplementary Material

Supplementary information
