# Supplementary material for: Myh6-driven Cre recombinase activates the DNA damage response and the cell cycle in the myocardium in the absence of loxP sites
Source: Dis Model Mech. 2020 Dec 18;13(12):dmm046375. doi: 10.1242/dmm.046375 (PMC7758623; doi:10.1242/dmm.046375)
Supplement: Supplementary information [file dmm-13-046375-s1.pdf]

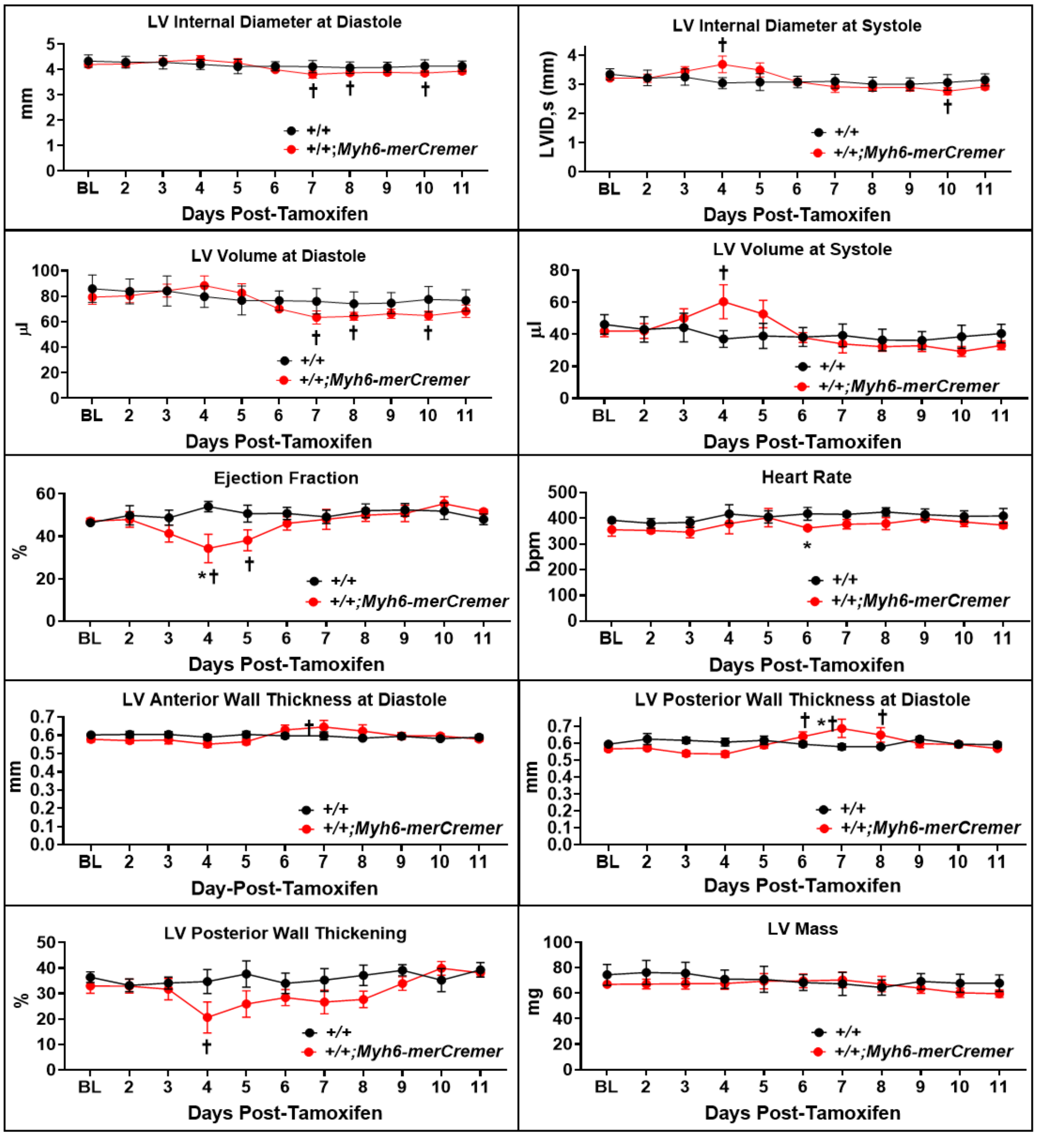

Figure S1. Echocardiographic assessments showing effects of activated merCremer in the absence of loxP sites. Adult 10-14 week-old naïve wild-type (+/+) mice (n=4), and mice (n=6) expressing a tamoxifen-induced merCremer-recombinase transgene (*+/+;Myh6-merCremer*), were injected with tamoxifen (40 mg/kg) on three consecutive days followed by echocardiographic assessment of the left ventricle. *P < 0.05 vs +/+; †P < 0.05 vs baseline value (day 0).

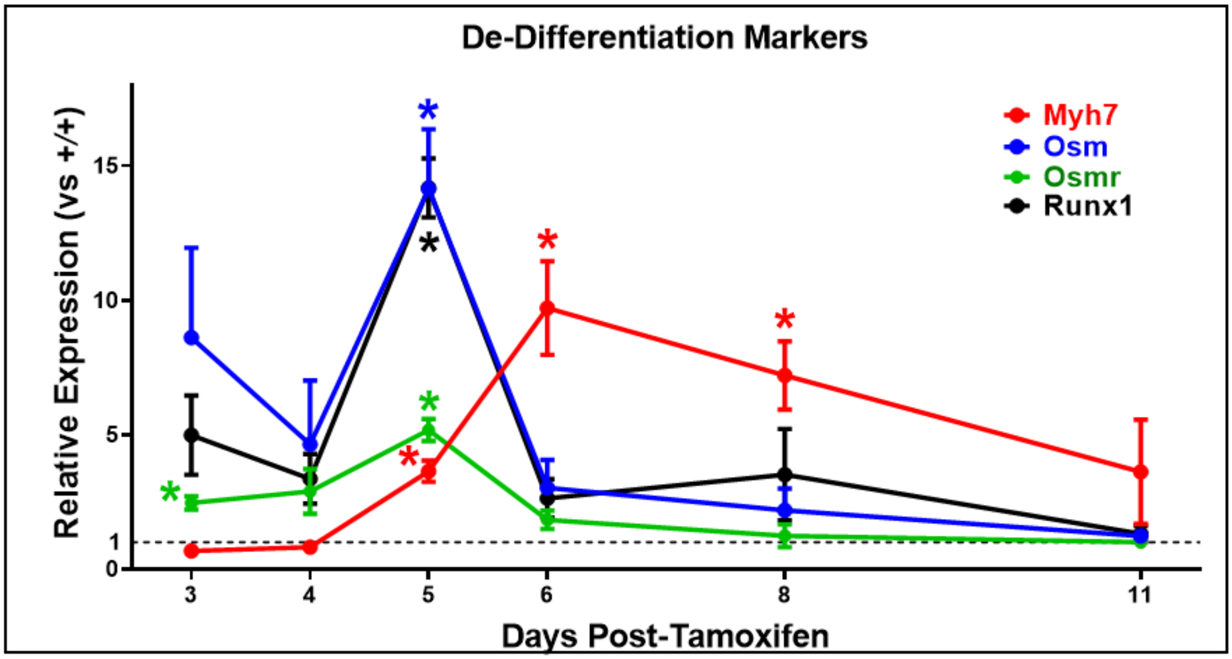

Figure S2. qPCR determinations show induction of de-differentiation marker genes following tamoxifen-induced activation of merCremer. Colored lines denote expression of each gene in *+/+;Myh6-merCremer* hearts, relative to expression in +/+ wild-type controls at Y=1 (broken line). Expression of each gene was normalized to *Gapdh*. N=3 hearts per timepoint; *P < 0.05 versus +/+.

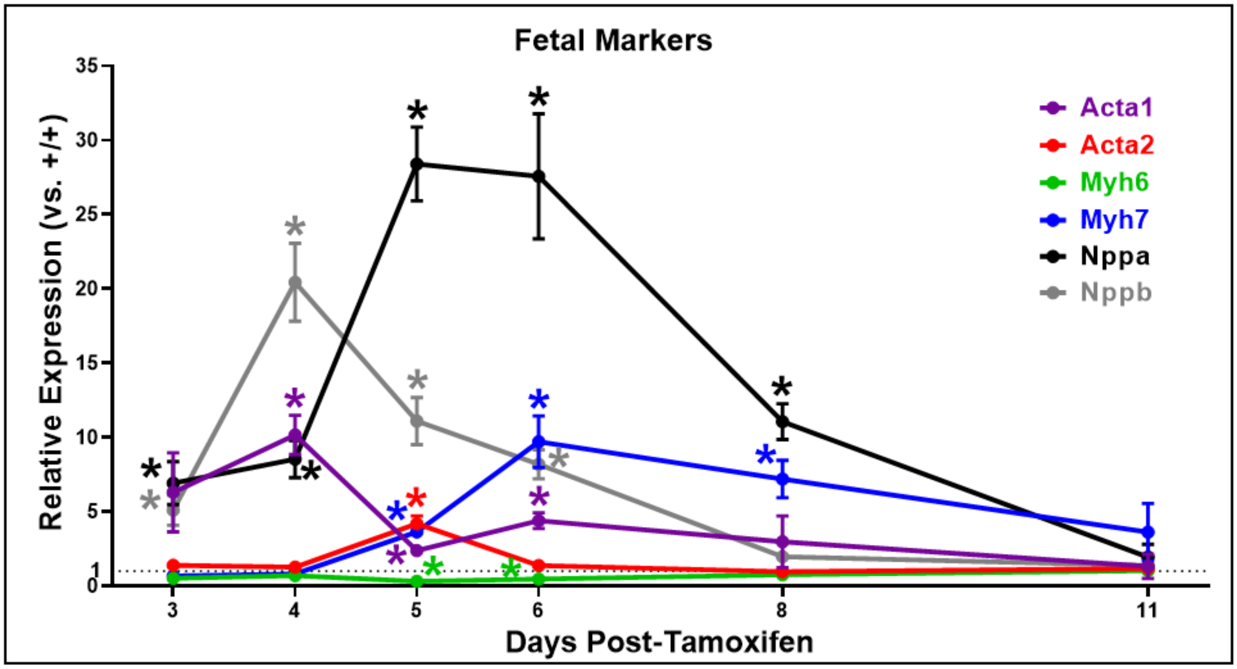

Figure S3. qPCR determinations show induction of fetal gene markers following tamoxifen-induced activation of merCremer. Colored lines denote expression of each gene in *+/+;Myh6-merCremer* hearts, relative to expression in +/+ wild-type controls at Y=1 (broken line). Expression of each gene was normalized to *Gapdh*. N=3 hearts per timepoint; *P < 0.05 versus +/+.

Disease Models & Mechanisms • Supplementary information

Table S1. ΔCq (relative to Gapdh) of DNA Damage Markers in +/+ Wild-type Controls & +/+;*Myh6-merCremer* Hearts (data are presented as means ±SEM)

**+/+**						
Gene	Days Post-Tamoxifen (N = 3 per timepoint)					
	3	4	5	6	8	11
Bax	7.54±0.16	7.14±0.30	7.67±0.07	7.16±0.18	7.32±0.13	7.06±0.14
Brca1	16.45±0.11	15.95±0.38	15.69±0.01	15.68±0.21	15.19±0.25	15.40±0.30
P53	8.71±0.10	8.70±0.37	9.50±0.20	8.84±0.37	8.45±0.21	8.36±0.23
Vcam1	8.20±0.28	7.92±0.29	8.49±0.10	7.91±0.27	7.87±0.08	7.61±0.16
Wee1	8.89±0.38	8.33±0.02	8.64±0.10	8.47±0.06	9.00±0.28	8.94±0.04
+/+;Myh6-merCremer						
Gene	Days Post-Tamoxifen (N = 3 per timepoint)					
	3	4	5	6	8	11
Bax	6.24±0.17	6.11±0.66	5.82±0.16	6.65±0.25	7.14±0.08	7.13±0.20
Brca1	14.22±0.44	13.15±1.44	10.82±0.21	13.10±1.01	13.51±0.70	14.91±0.25
P53	7.81±0.24	7.96±0.86	7.22±0.16	8.35±0.45	8.14±0.21	8.57±0.24
Vcam1	7.79±0.29	7.27±0.68	6.39±0.33	7.50±0.19	7.45±0.27	7.53±0.19
Wee1	8.61±0.33	8.42±0.36	7.89±.14	8.57±0.15	8.95±0.57	8.57±0.14

Disease Models & Mechanisms • Supplementary information

	Baseline		3 days-post-MI		10 days-post-MI		21 days-post-MI		28 days-post-MI	
	+/+ N=5	+/+;*Myh6-merCremer* N=5	+/+ N=5	+/+;*Myh6-merCremer* N=5	+/+ N=5	+/+;*Myh6-merCremer* N=5	+/+ N=5	+/+;*Myh6-merCremer* N=5	+/+ N=5	+/+;*Myh6-merCremer* N=5
LVAW d (mm)	0.62±0.03	0.62±0.03	0.79±0.02†	0.70±0.07	0.68±0.02	0.57±0.03	0.63±0.05	0.55±0.02	0.60±0.04	0.53±0.03
LVAW s (mm)	0.82±0.03	0.85±0.03	0.92±0.03	0.78±0.08	0.85±0.03	0.64±0.04*†	0.73±0.07	0.64±0.04†	0.70±0.05	0.61±0.03†
LVPW d (mm)	0.60±0.02	0.62±0.03	0.73±0.40	0.71±0.07	0.68±0.03	0.28±0.05	0.62±0.03	0.59±0.04	0.64±0.03	0.56±0.06
LVPW s (mm)	0.82±0.03	0.85±0.02	0.91±0.02	0.82±0.08	0.91±0.01	0.66±0.07*†	0.77±0.04	0.65±0.06†	0.82±0.04	0.68±0.08
LVID d (mm)	3.83±0.14	4.22±0.20	3.94±0.19	4.64±0.24	4.47±0.11	5.54±0.37*†	4.63±0.19	5.92±0.39*†	4.76±0.13†	6.02±0.37*†
LVID s (mm)	2.89±0.16	3.16±0.24	3.16±0.17	4.10±0.24	3.51±0.06	5.09±0.42*†	3.86±0.22	5.45±0.43*†	4.05±0.15†	5.58±0.39*†
FS (%)	24.7±1.8	25.4±2.3	19.5±2.8	11.6±1.9*†	21.4±1.5	8.4±1.6*†	16.8±2.4†	8.4±1.5*†	14.9±2.2†	7.6±1.3†
LV vol; d (µl)	61.5±4.3	77.9±9.0	68.6±3.6	91.6±8.7	83.0±6.7	137.2±13.0*†	89.7±11.3	167.5±19.2*†	99.3±8.9	165.8±19.2*†
LV vol; s (µl)	37.0±2.7	46.5±5.4	49.6±2.1	66.4±6.5	60.7±4.5	117.3±13.1*†	65.2±8.2	145.8±19.4*†	74.3±6.7†	144.2±17.7*†
EF (%)	39.9±0.3	40.4±0.2	27.6±0.9†	27.6±0.5†	26.6±0.7†	15.3±2.2*†	27.2±0.5†	14.1±2.6*†	25.1±1.0†	13.2±2.0*†
MPI	0.33±0.02	0.34±0.01	0.45±0.02†	0.46±0.01†	0.36±0.03	0.49±0.02*†	0.43±0.04†	0.48±0.02†	0.47±0.03†	0.47±0.02†
HR (bpm)	370±10	374±11	439±4†	418±13	385±28	429±2	364±22	425±30	374±14	416±20

Table S2: Echocardiographic assessment of infarcted +/+ and +/+;*Myh6-merCremer* mice. *P<0.05 vs. +/+ and †P<0.05 vs. baseline (Day 0).

Table S3. Primers & Taqman Probe Kits		
**for PCR Genotyping**		
Transgene	Sequence	Amplicon (bp)
Myh6-merCremer	FWD 5'-ATACCGGAGATCATGCAAGC-3'	440 bp
	REV 5'-AGGTGGACCTGATCATGGAG-3'	
Cycling Details: 94ºC 5 min, then 35 cycles of 94ºC 30sec/61ºC 45sec/72ºC 45sec, then 72ºC 10 min		

**for Taqman qRT-PCR**	
**Gene Target**	**Thermo-Fisher catalog #**
**Normalizers**	
Gapdh	Mm99999915_g1
**Cell-Cycle Activation Markers**	
Ccna2 (Cyclin A2)	Mm00438063_m1
Ccnb1 (Cyclin B1)	Mm03053893_gH
Cdk1	Mm00772472_m1
Ccnd1 (Cyclin D1)	Mm00432359_m1
Ccnd2 (Cyclin D2)	Mm00438070_m1
Cdk4	Mm00726334_s1
**Cell-Cycle Inhibitors**	
Cdkn1a (p21)	Mm00432448_m1
Cdkn1b (p27)	Mm00438168_m1
**DDR Markers**	
Bax	Mm00432051_m1
Brca1	Mm00515386_m1
Trp53 (p53)	Mm01731290_g1
Vcam1	Mm01320970_m1
Wee1	Mm00494175_m1
**De-Differentiation Markers**	
Myh7	Mm00600555_m1
Osm	Mm01193966_m1
Osmr	Mm01307326_m1
Runx1	Mm01213404_m1

Table S4. Antibodies for Immunofluorescent Staining					
	Antigen	Manufacturer	Catalog #	Made in	Dilution
1º	5'-bromodeoxyuridine (BrdU)	Abcam	ab6326	rat	1:200
2º	goat anti-rat 594	Invitrogen	A-11007	goat	1:500
1º	phosphohistone H3 (pH3)	Millipore	06-570	rabbit	1:400
2º	goat anti-rabbit 594	Invitrogen	A-11037	goat	1:500
1º	Ki67	Invitrogen	14-5698-82	rat	1:250
2º	goat anti-rat 594	Invitrogen	A-11007	goat	1:500
1º	Cre-recombinase	Millipore	69050-3	rabbit	1:500
2º	goat anti-rabbit 594	Invitrogen	A-11037	goat	1:500
1º	cardiac-Troponin (cTnT)	Abcam	ab8295	mouse	1:200
2º	goat anti-mouse 488	Invitrogen	A-11029	goat	1:500

Disease Models & Mechanisms • Supplementary information
